# Impact of Molecular Diagnostics for Tuberculosis on Patient-Important Outcomes: A Systematic Review of Study Methodologies

**DOI:** 10.1371/journal.pone.0151073

**Published:** 2016-03-08

**Authors:** Samuel G. Schumacher, Hojoon Sohn, Zhi Zhen Qin, Genevieve Gore, J. Lucian Davis, Claudia M. Denkinger, Madhukar Pai

**Affiliations:** 1 McGill University Department of Epidemiology & Biostatistics, Montreal, Canada; 2 McGill International TB Centre, Montreal, Canada; 3 McGill University, Schulich Library of Science and Engineering, Montreal, Canada; 4 UCSF Pulmonary & Critical Care Medicine, San Francisco, United States of America; 5 Beth Israel Deaconess Medical Centre, Division of Infectious Disease, Boston, MA, United States of America; National Institute for Infectious Diseases (L. Spallanzani), ITALY

## Abstract

**Background:**

Several reviews on the accuracy of Tuberculosis (TB) Nucleic Acid Amplification Tests (NAATs) have been performed but the evidence on their impact on patient-important outcomes has not been systematically reviewed. Given the recent increase in research evaluating such outcomes and the growing list of TB NAATs that will reach the market over the coming years, there is a need to bring together the existing evidence on impact, rather than accuracy. We aimed to assess the approaches that have been employed to measure the impact of TB NAATs on patient-important outcomes in adults with possible pulmonary TB and/or drug-resistant TB.

**Methods:**

We first develop a conceptual framework to clarify through which mechanisms the improved technical performance of a novel TB test may lead to improved patient outcomes and outline which designs may be used to measure them. We then systematically review the literature on studies attempting to assess the impact of molecular TB diagnostics on such outcomes and provide a narrative synthesis of designs used, outcomes assessed and risk of bias across different study designs.

**Results:**

We found 25 eligible studies that assessed a wide range of outcomes and utilized a variety of experimental and observational study designs. Many potentially strong design options have never been used. We found that much of the available evidence on patient-important outcomes comes from a small number of settings with particular epidemiological and operational context and that confounding, time trends and incomplete outcome data receive insufficient attention.

**Conclusions:**

A broader range of designs should be considered when designing studies to assess the impact of TB diagnostics on patient outcomes and more attention needs to be paid to the analysis as concerns about confounding and selection bias become relevant in addition to those on measurement that are of greatest concern in accuracy studies.

## Introduction

Tuberculosis (TB) continues to take a massive toll on human health globally, causing 1.5 million deaths and 9 million new cases annually[1]. The World Health Organization (WHO) recently set ambitious targets in the End TB Strategy to eradicate TB globally, with accurate and rapid detection of TB and drug resistance as critical components of their strategy[2]. Although several new diagnostics are now available for TB, sputum smear microscopy continues to be widely used, despite its limited sensitivity and inability to detect drug-resistance[3]. Mycobacterial culture has high sensitivity but takes weeks or months to yield results, such that its impact on clinical decision-making and patient-important outcomes is often limited[4–7].

Newer molecular TB diagnostics (nucleic acid amplification tests, NAATs) have been shown to have good accuracy and can produce rapid results[8–10], characteristics which have led to their endorsement by the WHO[11]. However, as it has become apparent that higher accuracy does not necessarily translate into improved patient care, policy makers have begun to demand more direct evidence that improved diagnostics positively affect health or other outcomes that matter to patients[12–14]. Much uncertainty remains about how new tests should be implemented to maximize their impact, which patient-important outcomes should be measured, and which designs should be used to assess them[13,15].

Given the recent increase in research evaluating the impact of TB NAATs on patient-important outcomes and the growing list of TB NAATs expected to reach the market in coming years, there is a need to summarize the existing evidence and best practices on methodologies for evaluating the impact of TB diagnostics[16]. Various designs have been used to measure a wide range of patient-important outcomes, but no systematic review has been done of such studies and the specific methodological issues that arise in them.

In this systematic review we aim to critically assess the approaches that have been employed to measure the impact of TB NAATs on patient-important outcomes (such as time to treatment initiation or mortality) in adults with possible pulmonary TB and/or drug-resistant TB from currently available evidence. The specific objectives of the systematic review of available studies were (i) to develop a conceptual framework for relevant patient-important outcomes and to assess which outcomes have been measured; (ii) to outline which designs and methodologies might be employed to measure the impact of diagnostics on patient-important outcomes and to assess which ones have been utilized; (iii) to propose criteria for assessing risk of bias for each design based on sound epidemiological principles where such tools do not already exist and to assess risk of bias and quality of reporting.

## Methods

We drafted a protocol before commencing the review, following standard guidelines[17,18]. We then developed a conceptual framework to clarify how improved test performance may lead to improved patient health via intermediate outcomes[19–23]. We also specified a framework for classifying research designs that have been (or could be) used to assess impact, using standard classifications from the field of epidemiology, clinical and diagnostic research[24–26], economics, and social sciences[27], and included designs only recently[28,29] or never previously suggested to our knowledge. This framework provides structure to the review and points to designs that have not been used, and provides a basis for assessing risk of bias, because threats to validity may differ between designs.

### Eligibility Criteria

We restricted our review to adult pulmonary TB, including drug-resistant TB, because diagnosis of childhood and extrapulmonary TB pose special diagnostic challenges and warrant consideration of contextual factors and methodological issues outside the scope of this review. We focused on the WHO-approved TB NAATs, i.e. two Line Probe assays (the GenoType MTBDRplus and the Inno-LiPA Rif.TB, both referred to simply as LPAs from here on) and the Xpert® MTB/RIF assay (Xpert), because these tests are currently of greatest interest globally and are being implemented in many countries around the world. We included only peer-reviewed studies that measured at least one patient-important outcome, i.e. outcomes that directly reflect some improvement in the patient’s experience that may directly affect health (such as a more rapid diagnosis, more rapid initiation of treatment, reduced mortality etc.). While cost is an important factor for patients, this was outside of the scope of this review and studies focusing on cost were excluded. We did not exclude any study design and did not restrict based on region, setting, years, or language. We only considered studies that included primary data, and excluded meta-analyses and compartmental and decision-analytic modeling studies. Studies that provided diagnostic test accuracy only were excluded.

### Information Sources

We searched MEDLINE, EMBASE, Web of Knowledge and Cochrane CENTRAL through January 31, 2015. We also searched the metaRegister of Controlled Trials (mRCT) and the WHO International Clinical Trials Registry Platform to identify ongoing trials. The full electronic search strategy can be found in ‘S1 Appendix’. To identify additional studies, we reviewed reference lists of included articles and of systematic reviews on the diagnostic accuracy of NAATs, and contacted researchers at FIND, members of the Stop TB Partnership's New Diagnostics Working Group, and other experts on TB diagnostics.

### Study Selection

Two review authors (SGS, ZQ) independently assessed titles and abstracts identified by electronic literature searching to identify potentially eligible studies (screen 1). Any citation identified by either review author during screen 1 was selected for full-text review. Two authors (SGS, HS) independently assessed articles for inclusion using predefined inclusion and exclusion criteria (screen 2), with discrepancies resolved by discussion. We maintained a list of excluded studies by reason for exclusion.

### Data Collection Process and Data Items

We developed a standardized data extraction form using Google Forms (Google Inc., Mountain View, CA, USA) to minimize data-entry errors[30]. Two authors (SGS, HS) piloted and revised the form to improve clarity. They then independently extracted data on study design, key contextual factors, patient-important outcomes, results for these outcomes and on design-specific risks for bias on a quarter (6/25) of studies. For the remainder of the studies, one author (SGS) extracted the data and the second (HS) crosschecked all extracted items.

### Risk of Bias in Individual Studies

We assessed risk of bias of individual studies with component questions for risk of bias assessment dependent on the study design. For randomized controlled trials we used the Cochrane risk of bias tool [31]. For the other study designs there is currently no suitable validated tool for risk of bias assessment[32]. We therefore decided on methodological components separately for each study design that likely place a study at higher risk of bias. Our choices were informed by other existing tools for risk of bias assessment [33–35], approaches taken by health technology assessment units[36–38] as well as generally recognized epidemiological and statistical principles[24]. The design-specific questions and guidance for how to judge each item is available in ‘S2 Appendix’. We did not attempt any assessment of risk of bias across studies.

### Synthesis of Results

We summarized characteristics of included studies by design and index test. Because of heterogeneity at all levels of abstraction, we did not plan to provide a meta-analysis, but instead a narrative synthesis of results structured around the conceptual frameworks. We used descriptive statistics to summarize key characteristics that we abstracted, stratified by study design. We used vote counting to assess how often authors reported that use of a TB NAAT positively influenced a particular outcome. We displayed results from the bias risk assessment graphically by study design across included studies and for each individual study. We calculated binomial 95% confidence intervals if not reported. Data management and descriptive statistics were done using STATA (version 12, Stata Corp, College Station, Texas, USA).

## Results

### Study Selection

**Fig 1:**
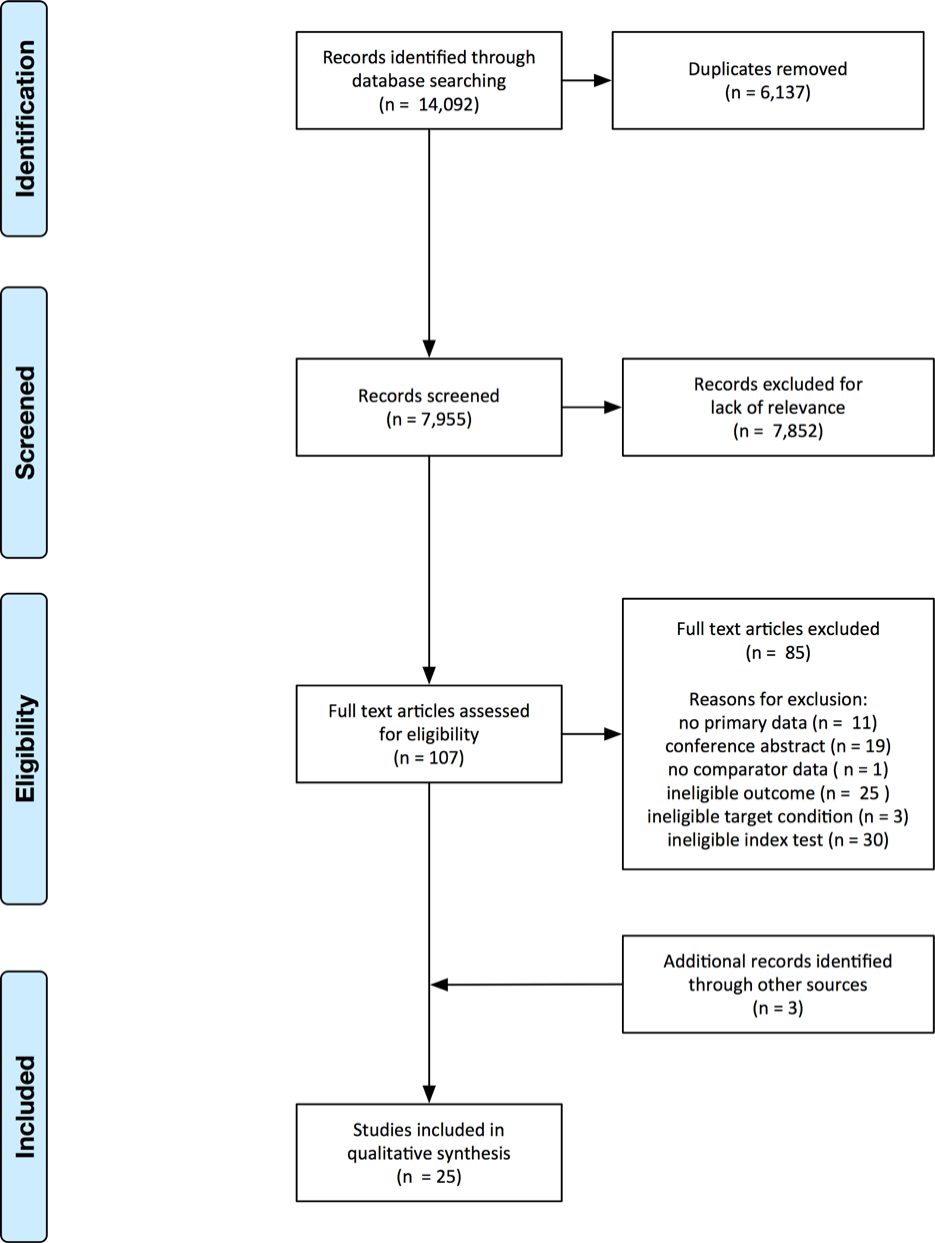
Study selection. Flow diagram of studies in the review. Note: Some studies had more than one reason for exclusion.

We screened the 7,995 abstracts that remained after removal of duplicates and identified 107 potentially eligible articles for which we obtained the full texts (Fig 1). We excluded 85 articles for reasons listed in Fig 1. We identified three additional studies, for a total of 25 included studies.

### Study Characteristics and Contextual Factors

Of 25 included studies (study characteristics in ‘S3 Appendix’), eleven (44%) were done in South Africa or involved a study site in South Africa as part of a multi-center study [39–48]. Most studies were carried out in urban hospitals (56%, n = 14[29,46,49–59] or urban primary care centers (32%, n = 8[39,40,42,43,47,48,60] with chest radiography reported to be available for TB diagnosis in almost three-quarters of studies (72%, n = 18) [29,40,43,44,46–55,58–60]. Of the 22 studies providing results from human immunodeficiency virus (HIV)-testing, 12 (55%) reported HIV prevalence greater than 50%[39,40,42,43,45–49,55,59]. Losses to follow-up before treatment initiation were reported in 10 studies and ranged from 7% to 39%[39,40,42,46–48,55,56,59]. The proportion of patients treated empirically (i.e. without microbiological confirmation) was reported in 12 studies and ranged from 10% to 69%[39,40,44,46–48,52,55,58–60].

Samples were transported for off-site testing in a laboratory in a different location from where patient care took place in 12 studies (48%)[29,40–43,45,46,49,51,54,55,60], and were tested on-site in the other 12[39,44,47,48,50,52,53,56,58,59] (location was unclear in the one remaining study) [57]. Of the 18 studies evaluating Xpert—a test yielding results within 2h—only four (22%)[48,53,58] attempted to implement the test embedded within a point-of-care testing program, where the goal is same-day treatment initiation. No other co-interventions aimed at ensuring or improving the continuum of care—from patient screening, through diagnostic testing, providing the TB or alternative diagnosis, ensuring linkage to care (avoiding loss to follow-up), initiating TB or alternative treatment, providing support for treatment adherence and ensuring successful treatment outcomes—were reported.

### Patient-Important Outcomes

**Fig 2:**
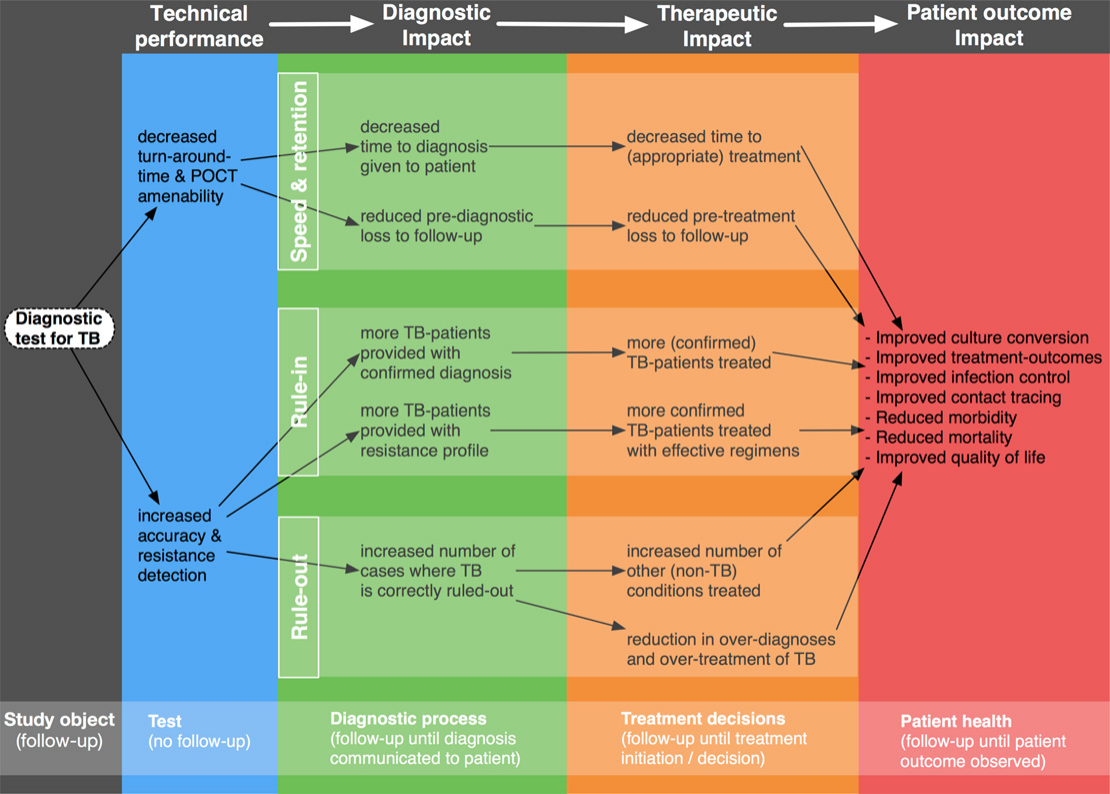
Conceptual framework of outcome measures. Conceptual framework outlining the pathways through which improved TB diagnostics may lead to improved patient outcomes.

The conceptual framework of the main pathways through which new TB diagnostics may affect patient health outcomes is shown in Fig 2. For clarity, and for consistency with what has been suggested previously[61], we differentiate four categories of outcome measures based on their object of study and required follow-up as indicated at the bottom of the figure: (i) measures of technical performance, (ii) measures of diagnostic impact, (iii) measures of therapeutic impact and (iv) measures of impact on patient outcomes. Definitions and examples of these four categories of outcome measures are provided in Table 1.

**Table 1:** Definitions and examples of categories of outcome measures.

Outcome measure	Definition	Examples
**Technical performance**	Outcome measures relating to the technical performance characteristics of the test.	Diagnostic accuracy, laboratory turn-around-time
**Diagnostic impact**	Outcome measures relating to how the test affects diagnosis and diagnostic processes.	Time to diagnosis, number of confirmed diagnoses provided
**Therapeutic impact**	Outcome measures relating to how the test affects treatment decisions.	Time to treatment, number of patients placed on appropriate therapy
**Patient outcome impact**	Outcome measures relating to patient health and/or quality of life.	TB treatment outcomes, mortality.

**Fig 3:**
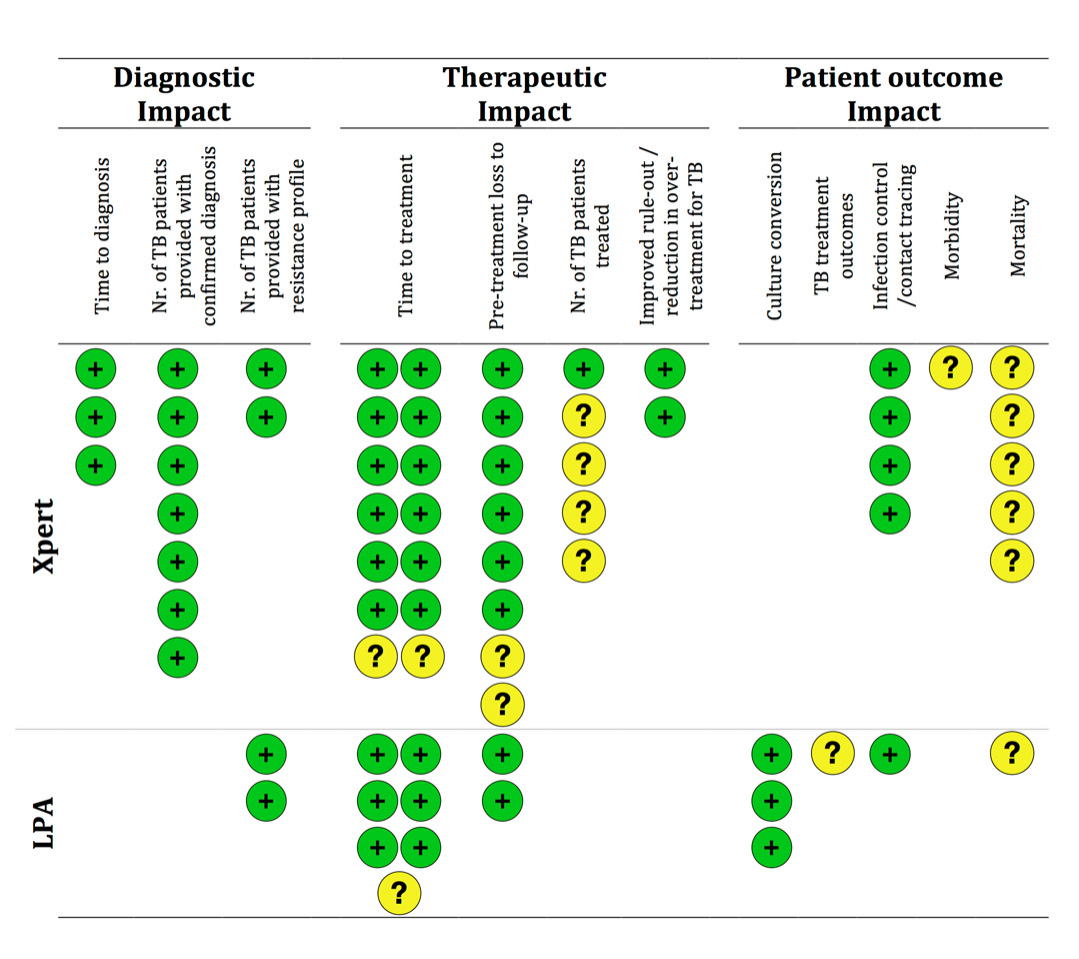
Reporting and vote counting of results on the different outcome measures. Each circle represents one study. Green circles represent a study finding that the TB NAAT improved the outcome, yellow circles represent a study with inconclusive findings where confidence intervals of the effect estimate included clinically relevant improvements or where confidence intervals were not provided and raw data for re-calculation was not accessible from the manuscript. Note: One study assessed both Xpert and LPA and is accounted for in both the upper section on Xpert and the lower section on LPA; some outcomes shown in Fig 2 were not reported in any study

Measures of technical performance (e.g. test accuracy) were often reported but not the focus of this review. The frequency of reporting of the various patient-important outcomes across included studies is shown in Fig 3. The most common measure of diagnostic impact was the ‘number of patients with confirmed diagnosis’ (28%, n = 7). We found that most studies included at least one measure of therapeutic impact with ‘reduced time to treatment initiation’ representing by far the most commonly used outcome measure overall (84%, n = 21), followed by ‘reduced loss to follow-up’ (36%, n = 9). Only about half of studies (n = 13) reported on a measure of patient outcome impact, mostly on mortality (24%, n = 6) and outcomes related to infection control or contact tracing (20%, n = 5).

No studies assessed the potential effects on increasing the number of diagnoses of other respiratory diseases, the number of patients treated with an effective regimen or health-related quality of life measures.

While included studies universally (100%, 14/14) showed benefit of the TB NAATs on the assessed measures of diagnostic impact and none showed any harm, only about three quarters (76%, 29/38) of measures of therapeutic impact and only half (50%, 8/16) of measures of impact on patient outcomes were shown to improve. None (0%, 0/7) of the studies was able to show a benefit on morbidity or mortality. However, point-estimates for changes in mortality and other measures of patient outcome impact often suggested improvements in the TB NAAT arm, but since these outcomes are relatively rare, confidence intervals were wide such that a “null-effect” could not be excluded. Unfortunately this was frequently interpreted as “no difference between arms” but wide intervals that include the null should not be interpreted as evidence favoring the null hypothesis[62,63]. For example, if a mortality-reduction of 25% or more is considered important from a clinical or public health standpoint, all findings on this outcome were inconclusive, rather than proving the absence of any relevant effect.

### Designs to Assess Impact of Diagnostic Tests

**Fig 4:**
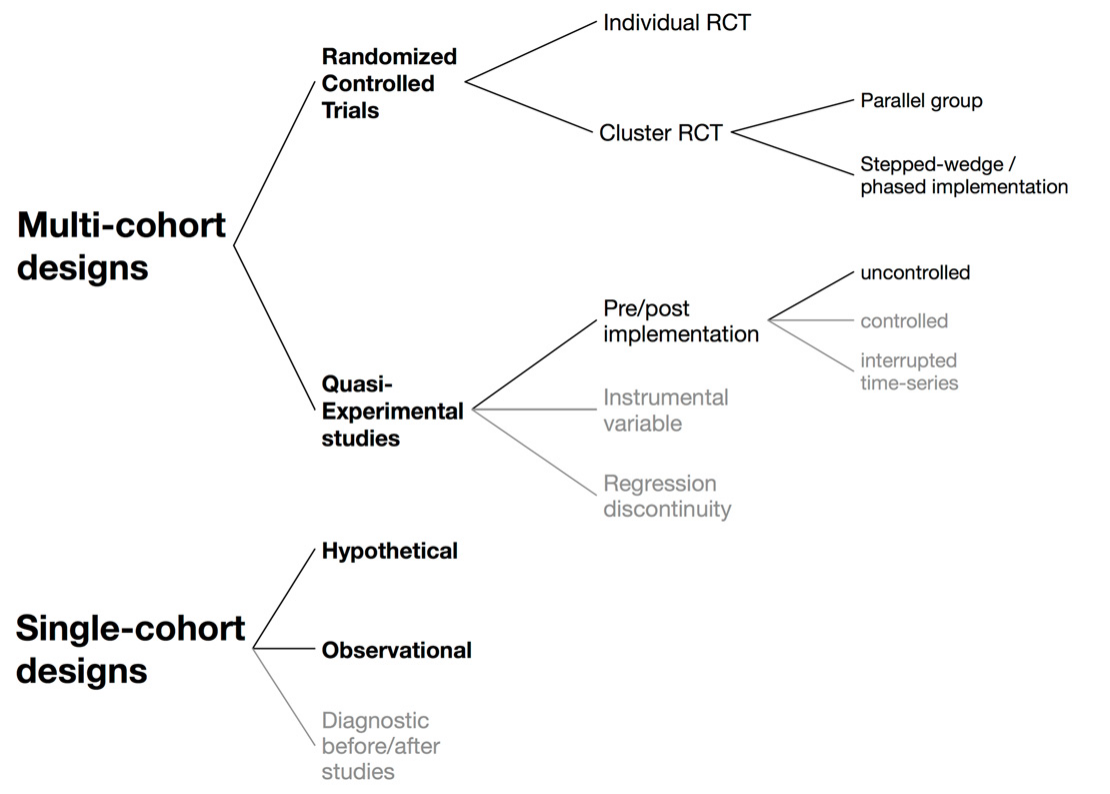
Design options to study the impact of TB diagnostics on patient health outcomes. Designs that have not been used in any of the studies included in this review are shown in grey. Of note, quasi-experimental studies are not typically described in epidemiological textbooks but are popular among economists and other social scientists: the basic idea of these designs is to try to make causal inference by exploiting some source of exogenous variation that acts similar to randomization. The three designs listed here may appear to be quite different but have the common feature that the type of exposure/test is neither the choice of the study participants (as in traditional cohort studies) nor assigned by the investigator (as in randomized trials) but determined through some exogenous factor. Pre/post implementation studies, where ‘time’ represents this exogenous factor, were the only quasi-experimental design used in the included studies.

The classification of design options as used in this review is shown in Fig 4. We first differentiate two major design architectures: (i) multi-cohort designs, i.e. designs that involve two (or more) cohorts, each exposed to only a single test or testing algorithm and (ii) single-cohort designs, i.e. designs that involve a single cohort exposed to multiple tests simultaneously. We then further differentiate important sub-types of these and brief descriptions for each design with references for further details in Table 2.

**Table 2:** Descriptions of design options to study the impact of TB diagnostics on patient-important outcomes with references on methodological issues.

Design category (and methodological references)	Design sub-type (and methodological references)	Description	References of studies included in the review
**Randomized Controlled Trials (RCTs)** [25,64]	**Individual RCT** [65]	Individuals are randomized to either receive or not receive the intervention	[55,66]
	**Cluster RCT** [25,67–70]	Parallel group: Clusters are randomized to either receive or not receive the intervention throughout the entire study period	[39,40]
		Stepped-wedge: The sequence in which clusters receive the intervention is randomized such that all clusters receive it by the end of the study (also called phased-implementation trial)	[60]
**Quasi-Experimental studies** [23,27,71–73]	**Pre/post implementation** [74–76]	Uncontrolled: Two cohorts are compared between two different time periods: the *pre* cohort receives the usual care during the baseline period and the *post* cohort receives the intervention during a subsequent and distinct time period.	[41–45,51,56,57,59]
		Controlled: Like ‘Uncontrolled pre/post implementation’ but including a contemporary control cohort that receives the usual care during both the *pre* and the *post* period, also called ‘difference in differences design’.	none
	**Interrupted time-series** [77–80]	Multiple measurements over time before and after implementation of the intervention analyzed using segmented regression or ARIMA models.	none
	**Instrumental variable** [81–85]	The effect of the intervention on the outcome is captured through another variable (the “instrument”) that affects the outcome only by affecting the intervention and does not share any causes with the outcome.	none
	**Regression discontinuity** [86–89]	The effect of the intervention on the outcome can be estimated if individuals receive the intervention based on whether they are above or below some threshold value on a continuous variable.	none
**Single-cohort designs**	**Hypothetical studies** [28]	A single cohort receives both baseline tests and the index test but results from the index tests are not used for patient management. “Hypothetical” changes in patient-important outcomes—had results been available to doctors—are estimated using a combination of study data, assumptions and potentially data from other studies.	[29,53,58,90]
	**Observational studies**	Inferences about the effect of the index test on patient-important outcomes are attempted based on a single cohort receiving both baseline tests and the index test with both being used for patient management.	[46–50,52,54]
	**Diagnostic before/after studies** [61,91–94]	Inferences about the effect of the index test on patient-important outcomes are attempted based on comparisons of the pre-test management plan (i.e. planned patient management before availability of index test results) and post-test management plan.	none

**Fig 5:**
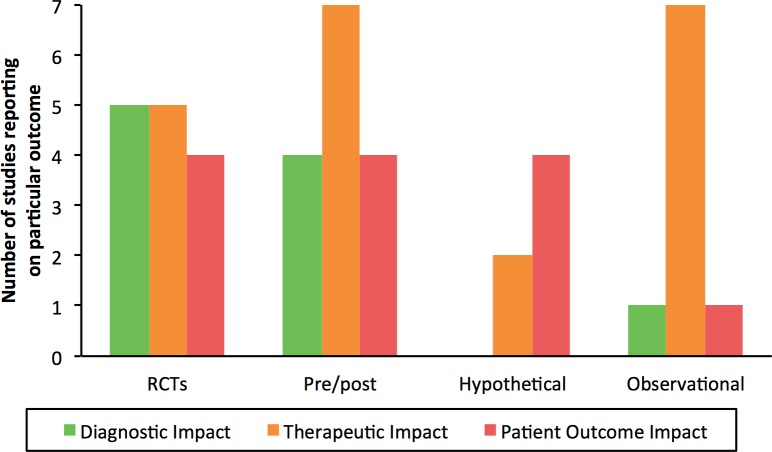
Frequency of studies reporting on one or several outcomes within the three outcome categories by study design.

As shown in Table 2, we identified two individually-randomized trials, two parallel-arm cluster-randomized trials and one stepped-wedge cluster-randomized trial. There were nine studies using a quasi-experimental design, all of which were uncontrolled pre/post implementation studies. We also found four single-cohort hypothetical studies and seven single-cohort observational studies but no diagnostic before/after studies. Authors did not provide any name for their study design in three studies and 13 different names were suggested by study authors for the remaining 17 non-randomized studies; we classified all 20 non-randomized studies into one of three study designs (Table 2). RCTs (randomized controlled trials) tended to assess outcomes across the whole range (i.e. from diagnostic impact to patient outcome impact) while pre/post and observational studies mostly assessed therapeutic impact and hypothetical studies mostly patient outcomes (Fig 5).

### Risk of Bias within Studies

**Fig 6:**
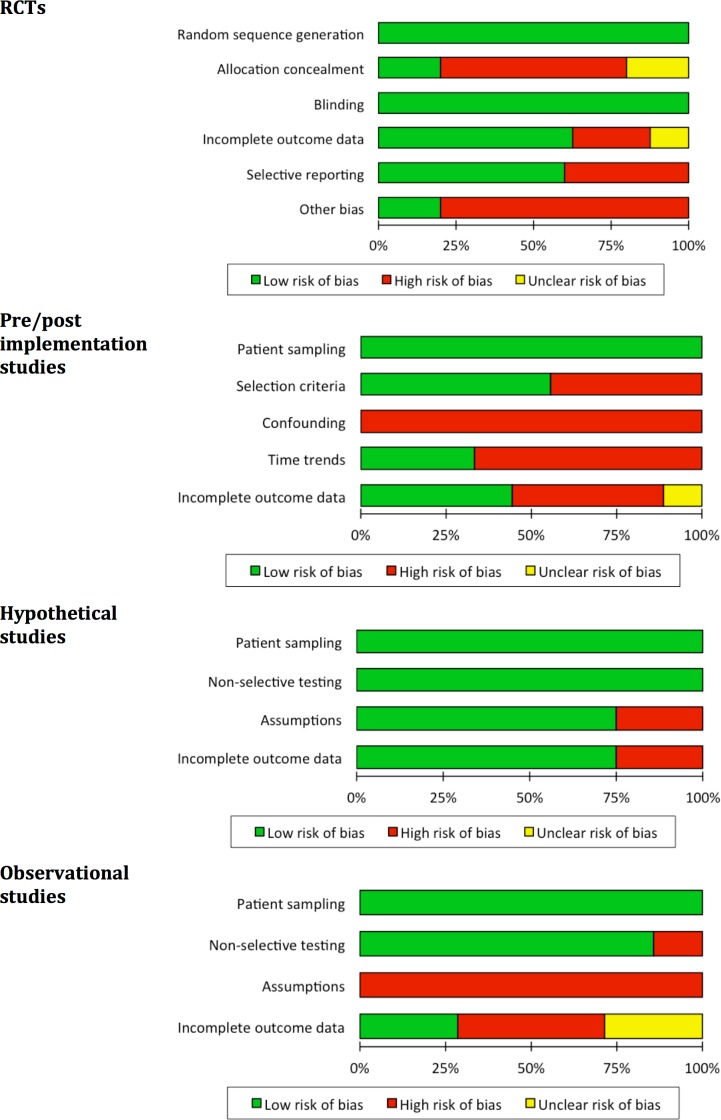
Assessment of risk of bias for each study design across included studies. Review authors’ judgments about each domain presented as percentages across the 25 studies, separately by design. Judgments were based on criteria outlined in ‘S2 Appendix’.

Results from the assessment of risk of bias are shown separately for each study design across included studies in Fig 6 and for each individual included study in ‘S4 Appendix’. For all designs incomplete outcome data was more common for measures of patient outcome impact than for outcomes further upstream in the causal chain. Where outcome data was missing, regression adjustment, imputation or sensitivity analyses were rarely attempted and this may have led to bias in some studies[95–97].

For all randomized trials, blinding of physicians to what test was done was impossible since knowing which test was done is part of the intervention itself. For example, the Xpert test has higher sensitivity than smear microscopy (and also produces RIF resistance results) and physicians must be allowed to take this into account when deciding about patient management. While outcomes between patients may therefore be different due to lack of blinding this was not judged to be a source of bias but rather the mechanism through which the intervention had an effect. Outcome measurement could theoretically have been influenced by the lack of blinding but this was deemed unlikely to cause bias of important magnitude. Overall, the lack of blinding was therefore judged not to put studies at increased risk of bias. Allocation concealment was impossible for cluster RCTs since once a cluster had been assigned to one of the intervention arms, allocation of this cluster was fixed and therefore unconcealed for the remainder of the study. However, if this had led to selection bias one would expect to see relevant covariate-imbalance between the intervention arms–this was not observed and therefore bias of important magnitude from this source is relatively unlikely.

For pre/post implementation studies, changes of patient eligibility criteria that occurred concurrently with the change of test used in the *pre* versus the *post* period were an important threat to validity. This was usually the result of a policy broadening eligibility for drug-resistance testing from a high-risk group in the *pre* period to all patients being evaluated for TB in the *post* period, implemented alongside the introduction of the TB NAAT. The resulting selection bias may be hard or impossible to address analytically. Temporal trends (usually changes in quality of care or care delivery) pose another potential risk of bias and study authors sometimes discussed this. A figure explaining how these mechanisms lead to lack of exchangeability and how this could potentially be addressed analytically is shown in ‘S5 Appendix’. Only three studies addressed the potential for confounding by providing either justification for why outcomes were likely unaffected or additional analysis. A table comparing pre/post cohorts was often shown but attempts to address existing covariate imbalances, e.g. via regression, were rare. If adjustment was attempted, methods were often described in insufficient detail (e.g. no explanation how continuous covariates were modeled[98,99]) and strategies for model selection were either not described or a method known to be prone to bias was used (e.g. inclusion based on p<0.05) [100–103]. As mentioned earlier, controlled pre/post implementation studies (and extensions via matched cohort designs[104]) were not identified, although such designs are preferable to protect the robustness of estimates from bias related to temporal trends.

Since in single-cohort studies comparisons are made *within* individuals, confounding and selection bias are not the main concern. The challenge for these designs lies in the assumptions one needs to make to draw valid conclusions about the effect of the index test. For the single-cohort observational studies, assumptions remained implicit and were often not justified. For example, patients testing negative on smear microscopy but positive with the TB NAAT were usually assumed to have received TB therapy *due to* the TB NAAT; however, this implicitly assumes that none of these patients would have been treated empirically, which is unlikely to hold true in most settings, leading to overestimates in the value of the test in placing more patients on therapy. In contrast, in Hypothetical studies assumptions were made explicit and risk of bias was overall low. This may in part reflect the fact that authors of Hypothetical studies were explicit in their aim to estimate causal effects of TB NAATs, while authors of observational studies were often simply aiming to provide some description of the use of these tests.

## Discussion

In this systematic review of the impact of molecular tuberculosis diagnostics on patient-important outcomes, we describe numerous challenges that may arise when choosing outcomes and designs. We describe the options that exist, the threats to validity that come with each choice, and make some suggestions about how to further raise methodological standards in design, analysis, and interpretation of results.

We found that most of the evidence on patient-important outcomes comes from a small number of settings with a particular epidemiological and operational context. Therefore, general conclusions about “the impact of TB NAATs” should be made with caution. The settings are not necessarily representative of the global TB epidemiology but may reflect the settings where research is most feasible due to availability of trained personnel, expertise and interest in research methodology, and beliefs about where the impact of the evaluated tests may be greatest.

Few studies assessed new tests implemented as part of point-of-care testing programs[105] and none aimed at ensuring a continuum of TB care. The importance of the cascade of care has been described extensively in the HIV literature[106] and it is becoming apparent that similar challenges in delivering services in a timely and reliable sequence exist in TB programs[107] and in point-of-care testing in global health in general[108]. Future studies and real-world implementation plans may need to take a more patient-centered approach in order for novel tests to reach their full potential in terms of improving patient and population health.

Included studies looked at a large variety of outcomes but “time to treatment initiation” was by far the most common one. Most studies showed benefit of TB NAATs and studies that did not show benefit were usually inconclusive (rather than affirming true absence of benefit) although this was generally poorly reported. Effect estimates should always be accompanied by Confidence Intervals[109] and resampling methods (such as the non-parametric bootstrap[110]) may be used if simple procedures to obtain them are not directly available in software packages, as is the case for changes in time to diagnosis or morbidity.

**Fig 7:**
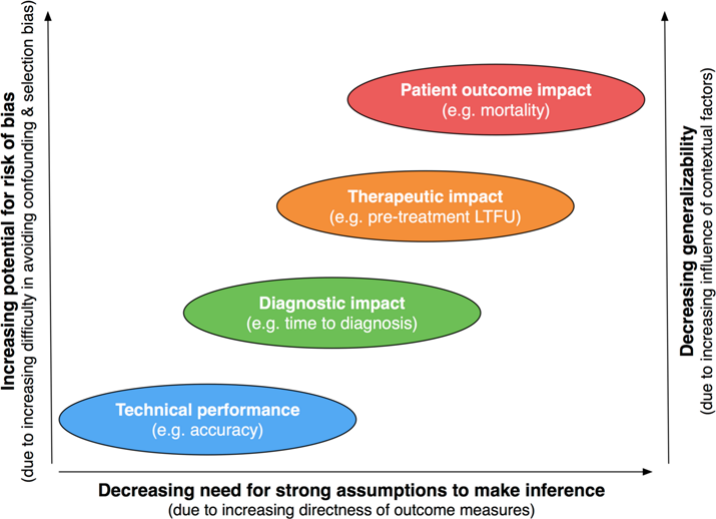
Directness of outcome measures, risk of bias and generalizability. Studies evaluating outcomes that provide very direct evidence for impacting patient outcomes may be–on average–more prone to confounding and selection bias and may lead to results that are less easily generalizable. The risk of confounding is likely increased because the number of covariates that have an influence on downstream outcomes (for which balance between compared cohorts needs to be ensured) increases. The risk of selection bias increases because the required length of follow-up increases as one assesses further downstream outcomes. Generalizability of specific estimates may become increasingly questionable because the role of contextual factors that vary from setting to setting also have increasing influence on the further downstream outcomes. In contrast, studies providing only very indirect evidence may have lower risk of bias but require much stronger assumptions if we try to extrapolate from their findings to make statements about downstream patient outcomes. In general it is therefore important to take both risk of bias and applicability into account to come to an overall conclusion about the likely impact of diagnostic tests on patient outcomes.

There is a trade-off when choosing outcome measures: while one may try to measure effects on patient health directly, this can come at greater risk of confounding, selection bias, and difficulties with generalizing findings to other settings; on the other hand one may resort to measure effects on patient health very indirectly, which can avoid or lower these risks but any conclusions about actual effects on patient health then require us to make a number of potentially untenable assumptions that are needed to such extrapolations (Fig 7). This is in fact a problem that health technology assessment units are routinely facing when trying to decide whether a new diagnostic test or screening program should be introduced[19,32,38,111]. High-quality evidence showing positive effects directly on patient outcomes is usually lacking which often leads assessors to use decision-analytic modeling as a way to integrate different pieces of evidence[28,112,113], and this approach may also be fruitful when assessing the value of TB diagnostics.

While randomized trials provide the strongest counterfactual, non-randomized studies will continue to play an important part in providing evidence on effects on patient-important outcomes. However, stronger non-randomized designs that can be based on routine data sources remain unused so far but may be well-suited for high-quality operational research studies and their use should be explored in future research. Missing outcome data was a relatively frequent problem and greater efforts should be made to address it using established methods[95–97]. Pre/post implementation studies suffered from selection bias and confounding, but either no attempt was made to address this analytically or attempts were methodologically problematic. Hypothetical trials can be an attractive option to estimate impact on patient outcomes by extrapolating more formally from test performance data based on explicitly stated and justified assumptions[28]. Single-cohort observational studies (as defined in our review) are probably not very suitable for inference on impact on patient outcomes but may still provide valuable insights about how tests are used in various settings.

We aimed to review the methodology of studies assessing two classes of TB NAATs, using a variety of study designs to measure a large number of different outcomes in settings with very different epidemiological and operational context. The multitude of these variables are both a strength and a weakness of our review as this allowed great breadth but limited depth in discussing methodological issues and their potential effects in making overall conclusions about the impact of the tests on patient-important outcomes.

We included non-randomized designs for which no validated tool to assess the risk of bias existed. The tools we used have not been validated and it is possible that relevant criteria were omitted or that other improvements could be made to our assessment of risk of bias. However, our tool was based on extensive search and review of the literature on assessing risk of bias in intervention studies, particularly those involving diagnostics[36–38] and included simple yes/no questions with explicit guidance on how to make judgments, as has been recommended for the development of new risk-of-bias tools[36].

We did not explicitly evaluate the potential for measurement bias because we did not feel that this was a big concern in general, which would need to be assessed for each individual study. However, we emphasize that the lack of a gold standard for TB (even mycobacterial culture has imperfect sensitivity), and the resulting reliance on clinical diagnosis, complicates the interpretation of outcomes such as the ‘number of *patients* put on treatment’, as this is only a proxy for the ‘number of *true TB patients* put on treatment’. Like any measurement error, use of this proxy leads to some degree of bias towards the nul[114]. Importantly though, it also has potential to introduce additional measurement bias towards the null because clinical diagnosis and empiric therapy is more common in the baseline arm (using smear microscopy), which likely leads to more *non-TB patients* put on TB treatment than in the TB NAAT arm. These are then counted as TB patients, thus seemingly improving the performance of the baseline arm, while actually representing in part over-treatment. It may be worthwhile to explore simple adjustments for this in sensitivity analyses in future studies[115,116], using a range of estimates of the accuracy of empiric therapy.

Our focus on adult pulmonary TB and WHO-approved NAATs was mostly a pragmatic decision. However, we believe that most of our conclusions will apply to other forms of TB as well as different infectious diseases and other types of tests. We focused on studies based on primary data but modeling studies have a key role in assessing patient (and population) impact as well. General advice on methodology for such studies exist [112,117–119] and an assessment of how they have been implemented in the case of point of care testing strategies for active tuberculosis[120] and for interferon-gamma release assays for latent TB infection[121] have been published.

## Conclusions

In conclusion, generating evidence on the impact of molecular TB diagnostics on patient-important outcomes is challenging and there is no simple or ideal choice for design or outcome. Choices will often be dictated by availability of routine data or limitations in funding to carry out primary data collection but an awareness of the trade-offs in choosing outcomes and designs will hopefully help make the best choices possible. Some designs that have the potential to yield strong evidence without requiring large-scale primary data collection have not been used to date and may have great potential for future research. Once data are collected, doing the best possible job during analysis is relatively inexpensive and should always be possible. As the analytic challenges are very different from those in accuracy research, including a methodologist during data analysis and ideally also early during study planning is advisable.

## Supporting Information

S1 Appendix Full electronic search strategy. (DOC)

S2 Appendix Design-specific risk of bias tools used and guidance for judgment. (DOCX)

S3 Appendix Study characteristics. (DOCX)

S4 Appendix Assessment of risk of bias for each individual included study. (PDF)

S5 Appendix Explanatory figure for Confounding and selection bias. (PDF)

S6 Appendix PRISMA 2009 checklist. (DOC)

## Abbreviations

TB: tuberculosis
NAAT: Nucleic Acid Amplification Tests
WHO: World Health Organization
LPA: Line Probe assay
Xpert: Xpert® MTB/RIF assay
MTB: Mycobacterium tuberculosis
RIF: Rifampicin
HIV: human immunodeficiency virus
RCT: randomized controlled trials
