# Supplementary material for: Impact of Molecular Diagnostics for Tuberculosis on Patient-Important Outcomes: A Systematic Review of Study Methodologies
Source: PLoS One. 2016 Mar 8;11(3):e0151073. doi: 10.1371/journal.pone.0151073 (PMC4783056; doi:10.1371/journal.pone.0151073)
Supplement: S1 Appendix — (DOC) [file pone.0151073.s001.doc]

Database: Embase Classic+Embase <1947 to 2015 January 29>

Search Strategy:

--------------------------------------------------------------------------------

1 exp tuberculosis/ (232802)

2 Mycobacterium tuberculosis/ (57525)

3 tuberculosis.mp. (265363)

4 tb.tw. (58072)

5 or/1-4 (301560)

6 nucleic acid amplification/ (4872)

7 molecular diagnosis/ (4583)

8 nucleic acid test*.tw. (886)

9 NAAT.tw. (312)

10 NAATs.tw. (208)

11 NAA.tw. (6639)

12 direct amplification.tw. (244)

13 transcription-mediated amplification.tw. (341)

14 RNA amplification*.tw. (629)

15 DNA amplification*.tw. (3916)

16 molecular assay*.tw. (2074)

17 molecular diagnos*.tw. (10596)

18 polymerase chain reaction*.tw. (186836)

19 PCR.tw. (431859)

20 PCRs.tw. (2951)

21 Xpert.tw. (463)

22 GeneXpert.tw. (200)

23 cepheid.tw. (340)

24 "MTB/RIF".tw. (218)

25 cobas.tw. (2822)

26 TaqMan.tw. (13731)

27 AMTD*.tw. (91)

28 MTD.tw. (6672)

29 Gen-Probe.tw. (753)

30 ligase chain reaction*.tw. (536)

31 LCx.tw. (2337)

32 line probe assay*.tw. (625)

33 LPA.tw. (4609)

34 LPAs.tw. (146)

35 AMTD*.tw. (91)

36 MTBDR*.tw. (176)

37 gMTBDR.tw. (1)

38 INNO-LiPA.tw. (580)

39 ProbeTec.tw. (117)

40 loopamp.tw. (12)

41 EXPAR.tw. (10)

42 LAMP.tw. (16871)

43 loop mediated amplification*.tw. (32)

44 Exponential Amplification Reaction*.tw. (9)

45 NALF.tw. (37)

46 nucleic acid lateral flow*.tw. (21)

47 (nucleic acid and amplification).tw. (5106)

48 (NAT or NATs).ti. (678)

49 (amplified and direct test*).tw. (142)

50 BD Probe.tw. (5)

51 Tec Direct.tw. (0)

52 or/6-51 (585569)

53 exp time/ (525789)

54 comparative effectiveness/ (8107)

55 exp "evaluation and follow-up"/ (1457223)

56 exp "treatment outcome"/ (979386)

57 exp morbidity/ (218643)

58 feasibility study/ (49946)

59 exp mortality/ (684720)

60 contact examination/ (2529)

61 exp infection control/ (80445)

62 cross infection/ (22558)

63 hospital information system/ (18032)

64 comparative study/ (743598)

65 intermethod comparison/ (179489)

66 exp survival/ (604552)

67 time to treatment/ (783)

68 exp diagnostic error/ (60382)

69 clinical decision making/ (16127)

70 medical decision making/ (65597)

71 decision making/ (140931)

72 exp "quality of life"/ (254950)

73 morbidity.tw. (327569)

74 feasibility.tw. (126601)

75 time.tw. (2635179)

76 mortality.tw. (637272)

77 outcome*.tw. (1189970)

78 conversion.tw. (164846)

79 follow-up.tw. (842102)

80 followup.tw. (32105)

81 decision*.tw. (270132)

82 impact.tw. (640283)

83 impacts.tw. (54478)

84 convert.tw. (24620)

85 delay*.tw. (437321)

86 adverse effect*.tw. (130415)

87 isolation.tw. (235688)

88 contact investigation*.tw. (572)

89 default.tw. (8909)

90 dropout*.tw. (8173)

91 drop-out*.tw. (6479)

92 empiric therapy.tw. (2227)

93 cure.tw. (94662)

94 failure*.tw. (695553)

95 relapse*.tw. (162955)

96 harm*.tw. (127568)

97 prevention.tw. (465774)

98 prevented.tw. (198315)

99 secondary case*.tw. (1603)

100 effectiveness.tw. (346458)

101 death*.tw. (738817)

102 undertreat*.tw. (5180)

103 under treat*.tw. (11600)

104 overtreat*.tw. (3330)

105 over treat*.tw. (1937)

106 adverse event*.tw. (116356)

107 adverse outcome*.tw. (18732)

108 undesirable effect*.tw. (3368)

109 patient centred.tw. (2978)

110 patient centered.tw. (6705)

111 contact tracing.tw. (1348)

112 contact examination*.tw. (140)

113 infection control.tw. (17400)

114 cross infection*.tw. (2817)

115 treatment fail*.tw. (26267)

116 recurrence.tw. (255869)

117 "point of care".tw. (8801)

118 survival.tw. (787636)

119 comparative stud*.tw. (102549)

120 "quality of life".tw. (206653)

121 qol.tw. (30987)

122 hrqol.tw. (10399)

123 or/53-122 (9669144)

124 5 and 52 and 123 (4402)

125 124 not (animal not human).sh. (4307)

***************************

1.

Gene expression profiles of ileal inflammatory bowel disease correlate with disease phenotype and advance understanding of its immunopathogenesis.

Ben-Shachar S., Yanai H., Baram L., Elad H., Meirovithz E., Ofer A., Brazowski E., Tulchinsky H., Pasmanik-Chor M., Dotan I.

Inflammatory Bowel Diseases. 19 (12) (pp 2509-2521), 2013. Date of Publication: November 2013.

[Journal: Article]

Publisher

Lippincott Williams and Wilkins (530 Walnut Street,P O Box 327, Philadelphia PA 19106-3621, United States)

<td colspan="">

Link to the Ovid Full Text or citation:

[Click here for full text options](http://ovidsp.ovid.com/ovidweb.cgi?T=JS&CSC=Y&NEWS=N&PAGE=fulltext&D=emedx&AN=2013749626)

Link to the External Link Resolver:

[Check SFX 1078-0998](http://mclink.library.mcgill.ca/sfx?sid=OVID:embase&id=pmid:&id=doi:10.1097%2F01.MIB.0000437045.26036.00&issn=1078-0998&isbn=&volume=19&issue=12&spage=2509&pages=2509-2521&date=2013&title=Inflammatory+Bowel+Diseases&atitle=Gene+expression+profiles+of+ileal+inflammatory+bowel+disease+correlate+with+disease+phenotype+and+advance+understanding+of+its+immunopathogenesis&aulast=Ben-Shachar&pid=<author>Ben-Shachar+S.%3BYanai+H.%3BBaram+L.%3BElad+H.%3BMeirovithz+E.%3BOfer+A.%3BBrazowski+E.%3BTulchinsky+H.%3BPasmanik-Chor+M.%3BDotan+I.<%2Fauthor><AN>2013749626<%2FAN><DT>Journal%3A+Article<%2FDT>)
