# Supplementary material for: Impact of Molecular Diagnostics for Tuberculosis on Patient-Important Outcomes: A Systematic Review of Study Methodologies
Source: PLoS One. 2016 Mar 8;11(3):e0151073. doi: 10.1371/journal.pone.0151073 (PMC4783056; doi:10.1371/journal.pone.0151073)
Supplement: S2 Appendix — (DOCX) [file pone.0151073.s002.docx]

**RCTs**

|  | **Was the allocation sequence adequately generated?** |
| --- | --- |
| Yes | The investigators describe a random component in the sequence generation process such as:   - Referring to a random number table; - Using a computer random number generator; - Coin tossing; - Shuffling cards or envelopes; - Throwing dice; - Drawing of lots; - Minimization*.     *Minimization may be implemented without a random element, and this is considered to be equivalent to being random. |
| No | The investigators describe a non-random component in the sequence generation process. Usually, the description would involve some systematic, non-random approach, for example:   - Sequence generated by odd or even date of birth; - Sequence generated by some rule based on date (or day) of admission; - Sequence generated by some rule based on hospital or clinic record number.     Other non-random approaches happen much less frequently than the systematic approaches mentioned above and tend to be obvious.  They usually involve judgement or some method of non-random categorization of participants, for example:   - Allocation by judgement of the clinician; - Allocation by preference of the participant; - Allocation based on the results of a laboratory test or a series of tests; - Allocation by availability of the intervention. |
| Unclear | - Insufficient information to permit judgment of ‘Yes’ or ‘No’ |
|  | **Was allocation adequately concealed?** |
| Yes | Participants and investigators enrolling participants could not foresee assignment because one of the following, or an equivalent method, was used to conceal allocation:   - Central allocation (including telephone, web-based and pharmacy-controlled randomization); - Sequentially numbered drug containers of identical appearance; - Sequentially numbered, opaque, sealed envelopes. |
| No | Participants or investigators enrolling participants could possibly foresee assignments and thus introduce selection bias, such as allocation based on:   - Using an open random allocation schedule (e.g. a list of random numbers); - Assignment envelopes were used without appropriate safeguards (e.g. if envelopes were unsealed or nonopaque or not sequentially numbered); - Alternation or rotation; - Date of birth; - Case record number; - Any other explicitly unconcealed procedure. |
| Unclear | - Insufficient information to permit judgement of ‘Yes’ or ‘No’. This is usually the case if the method of concealment is not described or not described in sufficient detail to allow a definite judgement – for example if the use of assignment envelopes is described, but it remains unclear whether envelopes were sequentially numbered, opaque and sealed. |
|  | **Was knowledge of the allocated interventions adequately prevented during the study?** |
| Yes | Any one of the following:   - No blinding, but the review authors judge that the outcome and the outcome measurement are not likely to be influenced by lack of blinding; - Blinding of participants and key study personnel ensured, and unlikely that the blinding could have been broken; - Either participants or some key study personnel were not blinded, but outcome assessment was blinded and the non-blinding of others unlikely to introduce bias. |
| No | Any one of the following:   - No blinding or incomplete blinding, and the outcome or outcome measurement is likely to be influenced by lack of blinding; - Blinding of key study participants and personnel attempted, but likely that the blinding could have been broken; - Either participants or some key study personnel were not blinded, and the non-blinding of others likely to introduce bias. |
| Unclear | Any one of the following:   - Insufficient information to permit judgement of ‘Yes’ or ‘No’; - The study did not address this outcome. |
|  | **Were incomplete outcome data adequately addressed?** |
| Yes | - No missing outcome data; - Reasons for missing outcome data unlikely to be related to true outcome (for survival data, censoring unlikely to be introducing bias); - Missing outcome data balanced in numbers across intervention groups, with similar reasons for missing data across groups; table of participants without missing outcome data presented, showing balance of relevant covariates - For dichotomous outcome data, the proportion of missing outcomes compared with observed event risk not enough to have a clinically relevant impact on the intervention effect estimate; - For continuous outcome data, plausible effect size (difference in means or standardized difference in means) among missing outcomes not enough to have a clinically relevant impact on observed effect size; - Missing data have been imputed using appropriate methods. |
| No | - Reason for missing outcome data likely to be related to true outcome, with either imbalance in numbers or reasons for missing data across intervention groups; - For dichotomous outcome data, the proportion of missing outcomes compared with observed event risk enough to induce clinically relevant bias in intervention effect estimate; - For continuous outcome data, plausible effect size (difference in means or standardized difference in means) among missing outcomes enough to induce clinically relevant bias in observed effect size; - Potentially inappropriate application of simple imputation. |
| Unclear | Any one of the following:   - Insufficient reporting of attrition/exclusions to permit judgement of ‘Yes’ or ‘No’ (e.g. number randomized not stated, no reasons for missing data provided); - The study did not address this outcome. |
|  | **Are reports of the study free of suggestion of selective outcome reporting?** |
| Yes | Any of the following:   - The study protocol is available and all of the study’s pre-specified (primary and secondary) outcomes that are of interest in the review have been reported in the pre-specified way; - The study protocol is not available but it is clear that the published reports include all expected outcomes, including those that were pre-specified (convincing text of this nature may be uncommon). |
| No | Any one of the following:   - Not all of the study’s pre-specified primary outcomes have been reported; - One or more primary outcomes is reported using measurements, analysis methods or subsets of the data (e.g. subscales) that were not pre-specified; - One or more reported primary outcomes were not pre-specified (unless clear justification for their reporting is provided, such as an unexpected adverse effect); - One or more outcomes of interest in the review are reported incompletely so that they cannot be entered in a meta-analysis; - The study report fails to include results for a key outcome that would be expected to have been reported for such a study. |
| Unclear | Insufficient information to permit judgment of ‘Yes’ or ‘No’. It is likely that the majority of studies will fall into this category. |
|  | **Was the study apparently free of other problems that could put it at a risk of bias?** |
| Yes | The study appears to be free of other sources of bias. |
| No | There is at least one important risk of bias. For example, the study:   - Had a potential source of bias related to the specific study design used; or - Stopped early due to some data-dependent process (including a formal-stopping rule); or - Had extreme baseline imbalance; or - Has been claimed to have been fraudulent; or - Had some other problem. |
| Unclear | There may be a risk of bias, but there is either:   - Insufficient information to assess whether an important risk of bias exists; or - Insufficient rationale or evidence that an identified problem will introduce bias. |

**Pre/post implementation studies**

|  | **Was a consecutive or random sample of patients enrolled?** |
| --- | --- |
| Yes | - authors state consecutive or random recruitment - AND patients’ disease (or resistance) status was unknown at enrollment |
| No | - authors state recruitment strategy other than random/consecutive - OR patients’ disease (or resistance) status was already known based at enrollment, e.g. only smear-positive patients |
| Unclear | - Insufficient information to permit judgment of ‘Yes’ or ‘No’ |
|  | **Were selection criteria the same across different cohorts?** |
| Yes | - there is no indication of differing selection criteria across the different cohorts, e.g. if recruitment was prospectively done as part of a research project that recruited patients based on rigorous inclusion criteria |
| No | - there is strong reason to believe or clear evidence of differing selection criteria across the different cohorts, e.g. due to changes in policy for test eligibility alongside introduction of a new test in routine practice |
| Unclear | - Insufficient information to permit judgment of ‘Yes’ or ‘No’ |
| Yes | **Was exchangeability assessed and addressed?** |
| Yes | - assessed (e.g. table comparing baseline differences between groups) and lack of exchangeability addressed (e.g. using regression adjustment) - OR assessed and groups found to be exchangeable - NOTE: ideally the assessment of exchangeability would have to be based on the outcome of interest, at the minimum balance of some covariates should have been assessed for a given outcome to judge this question as “yes” |
| No | - not assessed - OR assessed, lack of exchangeability found but not addressed - OR assessed, lack of exchangeability found and adjustment insufficient/inadequately implemented (e.g. key known confounders omitted, model selection procedure used that is known to lead to bias such as based on p<0.05) |
| Unclear | - Insufficient information to permit judgment of ‘Yes’ or ‘No’ |
|  | **Were time trends assessed and addressed?** |
| Yes | - assessed (e.g. time trends assessed either over study period or before or after study period) and no important trends found - OR assessed, important trends found and analytically addressed - OR assessed, important trends found and effects of this clearly discussed |
| No | - not assessed - assessed, important trends found but not analytically addressed or discussed |
| Unclear | - Insufficient information to permit judgment of ‘Yes’ or ‘No’ |
|  | **Were incomplete outcome data adequately addressed?** |
| Yes | - No missing outcome data; - Reasons for missing outcome data unlikely to be related to true outcome (for survival data, censoring unlikely to be introducing bias); - Missing outcome data balanced in numbers across intervention groups, with similar reasons for missing data across groups; table of participants without missing outcome data presented, showing balance of relevant covariates - For dichotomous outcome data, the proportion of missing outcomes compared with observed event risk not enough to have a clinically relevant impact on the intervention effect estimate; - For continuous outcome data, plausible effect size (difference in means or standardized difference in means) among missing outcomes not enough to have a clinically relevant impact on observed effect size; - Missing data have been imputed using appropriate methods. |
| No | - Reason for missing outcome data likely to be related to true outcome, with either imbalance in numbers or reasons for missing data across intervention groups; - For dichotomous outcome data, the proportion of missing outcomes compared with observed event risk enough to induce clinically relevant bias in intervention effect estimate; - For continuous outcome data, plausible effect size (difference in means or standardized difference in means) among missing outcomes enough to induce clinically relevant bias in observed effect size; - Potentially inappropriate application of simple imputation. |
| Unclear | - Insufficient information to permit judgment of ‘Yes’ or ‘No’ |

**Single-cohort observational & single-cohort hypothetical**

|  | **Was a consecutive or random sample of patients enrolled?** |
| --- | --- |
| Yes | - authors state consecutive or random recruitment - AND patients’ disease (or resistance) status was unknown at enrollment |
| No | - authors state recruitment strategy other than random/consecutive - OR patients’ disease (or resistance) status was already known based at enrollment, e.g. only smear-positive patients |
| Unclear | - Insufficient information to permit judgment of ‘Yes’ or ‘No’ |
|  | **Were all TB tests done on all participants?** |
| Yes | - all participants received or were at least supposed to receive all tests - AND only a small proportion ended up not getting all tests (e.g. because of failed tests) |
| No | - testing was done in a selective way - this may have happened e.g. because sicker patients getting a more rapid test, uninsured poor patients getting a cheaper test but should be rated as ‘No’ even if reasons are not provided |
| Unclear | - Insufficient information to permit judgment of ‘Yes’ or ‘No’ |
|  | **Were assumptions relating to the attribution of management decisions as being due to test results clearly stated and justified?** |
| Yes | - assumptions clearly stated and justified, paying particular attention to assumptions about empiric therapy (e.g. for a patient testing smear-negative, Xpert positive and put on therapy, was it assumed that therapy would not have been started without the Xpert result) - justifications may be a policy about patient management or an algorithm that is usually adhered to |
| No | - assumptions stated but no justification given - assumptions not stated (left implicit) |
| Unclear | - Insufficient information to permit judgment of ‘Yes’ or ‘No’ |
|  | **Were incomplete outcome data adequately addressed?** |
| Yes | - No missing outcome data; - Reasons for missing outcome data unlikely to be related to true outcome (for survival data, censoring unlikely to be introducing bias); - Missing outcome data balanced in numbers across intervention groups, with similar reasons for missing data across groups; table of participants without missing outcome data presented, showing balance of relevant covariates - For dichotomous outcome data, the proportion of missing outcomes compared with observed event risk not enough to have a clinically relevant impact on the intervention effect estimate; - For continuous outcome data, plausible effect size (difference in means or standardized difference in means) among missing outcomes not enough to have a clinically relevant impact on observed effect size; - Missing data have been imputed using appropriate methods. |
| No | - Reason for missing outcome data likely to be related to true outcome, with either imbalance in numbers or reasons for missing data across intervention groups; - For dichotomous outcome data, the proportion of missing outcomes compared with observed event risk enough to induce clinically relevant bias in intervention effect estimate; - For continuous outcome data, plausible effect size (difference in means or standardized difference in means) among missing outcomes enough to induce clinically relevant bias in observed effect size; - Potentially inappropriate application of simple imputation. |
| Unclear | - Insufficient information to permit judgment of ‘Yes’ or ‘No’ |

**Diagnostic before/after studies**

|  | **Was a consecutive or random sample of patients enrolled?** |
| --- | --- |
| Yes | - authors state consecutive or random recruitment - AND patients’ disease (or resistance) status was unknown at enrollment |
| No | - authors state recruitment strategy other than random/consecutive - OR patients’ disease (or resistance) status was already known based at enrollment, e.g. only smear-positive patients |
| Unclear | - Insufficient information to permit judgment of ‘Yes’ or ‘No’ |
|  | **Were all TB tests done on all participants?** |
| Yes | - all participants received or were at least supposed to receive all tests - AND only a small proportion ended up not getting all tests (e.g. because of failed tests) |
| No | - testing was done in a selective way - this may have happened e.g. because sicker patients getting a more rapid test, uninsured poor patients getting a cheaper test but should be rated as ‘No’ even if reasons are not provided |
| Unclear | - Insufficient information to permit judgment of ‘Yes’ or ‘No’ |
|  | **Were assumptions relating to the attribution of management decisions as being due to test results clearly stated and justified?** |
| Yes | - assumptions clearly stated and justified, paying particular attention to assumptions about empiric therapy (e.g. for a patient testing smear-negative, Xpert positive and put on therapy, was it assumed that therapy would not have been started without the Xpert result) - justifications may be a policy about patient management or an algorithm that is usually adhered to |
| No | - assumptions stated but no justification given - assumptions not stated (left implicit) |
| Unclear | - Insufficient information to permit judgment of ‘Yes’ or ‘No’ |
|  | **Were data on pre-test management decisions reliably captured?** |
| Yes | - data on pre-test management decisions was recorded by asking treating physicians about their management plans after results from routine tests were available and before results from the index tests were available - OR data were reliably available from electronic medical records or charts (possible if results from the index test always arrive after the routine workup has been completed) AND were extracted applying consistent criteria |
| No | - retrospective “guessing” about the likely pre-test management plan without formal assessment |
| Unclear | - Insufficient information to permit judgment of ‘Yes’ or ‘No’, e.g. no description of how pre-treatment decision was obtained |
