# Supplementary material for: Impact of Molecular Diagnostics for Tuberculosis on Patient-Important Outcomes: A Systematic Review of Study Methodologies
Source: PLoS One. 2016 Mar 8;11(3):e0151073. doi: 10.1371/journal.pone.0151073 (PMC4783056; doi:10.1371/journal.pone.0151073)
Supplement: S3 Appendix — (DOCX) [file pone.0151073.s003.docx]

|  | **Design** | **Test** | **Testing** | **Population** | **Country** | **Country setting** | **Clinical setting** | **CXR avail.** | **HIV** | **Smear-Cult.+** | **Rif / MDR** | **Empiric therapy** | **LTFU** |
| --- | --- | --- | --- | --- | --- | --- | --- | --- | --- | --- | --- | --- | --- |
| Theron  (2013)^38^ | Ind. RCT | Xpert | POCT | TB suspects | Multiple* | Urban | Clinic | yes | 60% | 50% | 6% | 66% | 15% |
| Mupfumi  (2014)^39^ | Ind. RCT | Xpert | Off-site | ART-initiators | Zimbabwe | Urban | Hospital | yes | 100% | NR | 0% | 69% | 18% |
| Cox  (2014)^40^ | Parallel Cl. RCT | Xpert | On-site | TB suspects | South Africa | Urban | Clinic | NR | 60% | NR | 1% | 10% | 25% |
| Durovni  (2014)^41^ | St.-we. Cl. RCT | Xpert | Off-site | TB suspects | Brazil | Urban | Clinic | yes | 10% | NR | <2%* | 62% | NR |
| Churchyard  (2015)^42^ | Parallel cRCT | Xpert | Off-site | TB suspects | South Africa | Urban,  rural | Clinic | yes | 62% | NR | 4% | 35% | 15% |
| Boehme  (2011)^43^ | Pre/post | Xpert | On-site | TB suspects, MDR suspects | Multiple** | Urban | Mixed | yes | 19% | 37% | 24% | 13% | NR |
| Yoon  (2012)^44^ | Pre/post | Xpert | On-site | Hospitalized TB suspects | Uganda | Urban | Hospital | yes | 76% | 29% | NR | 15% | 10% |
| Naidoo  (2014)^45^ | Pre/post | Xpert | Off-site | MDR suspects (smear-pos./cult.-pos. for LPA) | South Africa | Urban | Clinic | yes | 53% | NR | NR | NR | NR |
| Cox  (2015)^46^ | Pre/post | LPA, Xpert | Off-site | Lab-confirmed RR-TB patients | South Africa | Urban | Clinic | NR | 74% | NR | NR | NR | 16% |
| Skenders  (2011)  ^47^ | Pre/post | LPA | unclear | Smear-pos. MDR suspects | Latvia | Urban | Hospital | NR | NR | NR | 28% | NR | NR |
| Hanrahan  (2012)^48^ | Pre/post | LPA | Off-site | Smear-pos. or culture-pos. MDR suspects | South Africa | unclear | Clinic | NR | 58% | NR | 12% | NR | NR |
| Jacobsen  (2012)^49^ | Pre/post | LPA | Off-site | Culture-conf. MDR patients | South Africa | Rural | Hospital | NR | 30% | 62% | 100% | NR | NR |
| Kipiani  (2014)^50^ | Pre/post | LPA | Off-site | Culture-conf. MDR patients | Georgia | Urban | Hospital | yes | 3% | 33% | 100% | NR | NR |
| Singla  (2014)^51^ | Pre/post | LPA | On-site | MDR suspects | India | Urban | Hospital | NR | NR | NR | 17% | NR | 39% |

**Table 1. Study characteristics of multi-cohort studies (RCTs and pre/post implementation studies).** CXR avail.=availability of chest radiography to treating physicians. HIV=prevalence of Human Immunodeficiency Virus infection in study cohort. Smear-Cult.+=proportion of culture-positive patients testing negative by smear-microscopy (proxy for paucibacillary disease). Rif/MDR=prevalence of Rifampin or MDR-resistance. Empiric therapy=proportion of treated patients without microbiological confirmation. LTFU=pre-treatment loss to follow-up. Ind. RCT=Individually Randomized Controlled Trial. Cl. RCT=Cluster Randomized Controlled Trial. St.-we. Cl. RCT=Stepped-wedge Cluster Randomized Controlled Trial. Pre/post=Pre/post implementation study. POCT=Point-of-care Testing. TB=Tuberculosis. ART=Anti-retroviral Therapy. MDR=Multidrug-resistant tuberculosis. smear-pos.=positive on sputum smear microscopy. cult.-pos.=positive by mycobacterial culture. RR-TB=Rifampin-resistant tuberculosis. NR=not reported.

* South Africa, Zimbabwe, Zambia, and Tanzania. ** South Africa, Peru, and India, Azerbaijan, Philippines, Uganda.

|  | **Design** | **Test** | **Testing** | **Population** | **Country** | **Country Setting** | **Clinical setting** | **CXR avai.** | **HIV** | **Smear-Cult.+** | **Rif / MDR** | **Empiric therapy** | **LTFU** |
| --- | --- | --- | --- | --- | --- | --- | --- | --- | --- | --- | --- | --- | --- |
| Chaisson  (2014)^52^ | Hypothetical | Xpert | On-site | Hospitalized TB suspects | USA | Urban | Hospital | NR | 30% | 11% | 0% | NR | NR |
| Sohn  (2014)^53^ | Hypothetical | Xpert | POCT | TB suspects | Canada | Urban | Hospital | yes | 2% | 72% | 4% | 24% | NR |
| Lippincott  (2014)^54^ | Hypothetical | Xpert | On-site | Hospitalized TB suspects | USA | Urban | Hospital | yes | 24% | 0% | 17% | NR | NR |
| Davis  (2014)^28^ | Hypothetical | Xpert | Off-site | TB suspects started on empiric therapy | USA | Urban | Hospital | yes | 8% | 15% | 23% | NA | NR |
| Balcells  (2012)^55^ | Observational | Xpert | Off-site | HIV-pos. TB suspects | Chile | Urban | Hospital | yes | 100% | 33% | 17% | NR | NR |
| Lyu  (2013)^56^ | Observational | LPA | Off-site | Lab-confirmed TB patients | South Korea | Urban | Hospital | yes | 1% | 58% | 14% | NR | NR |
| Van Rie  (2013)^57^ | Observational | Xpert | POCT | Smear-negative TB suspects | South Africa | Urban | Clinic | yes | 72% | 53% | NR | 54% | 18% |
| Hanrahan  (2013)^58^ | Observational | Xpert | POCT | TB suspects | South Africa | Urban | Clinic | yes | 69% | 76% | NR | 41% | 11% |
| Kwak  (2013)^59^ | Observational | Xpert | On-site | TB suspects in whom Xpert was done | South Korea | Urban | Hospital | yes | 1% | 60% | 11% | 27% | NR |
| Ramirez  (2014)^60^ | Observational | Xpert | On-site | TB patients | Spain | Urban | Hospital | yes | NR | 47% | NR | NR | NR |
| Cohen  (2014)^61^ | Observational | Xpert | Off-site | HIV-pos. TB suspects in whom Xpert was done | South Africa | Urban | Hospital | yes | 100% | NR | NR | 39% | 7% |

**Table 2. Study characteristics of single-cohort studies (hypothetical and observational).** CXR avail.=availability of chest radiography to treating physicians. HIV=prevalence of Human Immunodeficiency Virus infection in study cohort. Smear-Cult.+=proportion of culture-positive patients testing negative by smear-microscopy (proxy for paucibacillary disease). Rif/MDR=prevalence of Rifampin or MDR-resistance. Empiric therapy=proportion of treated patients without microbiological confirmation. LTFU=pre-treatment loss to follow-up. Hypothetical=Single-cohort hypothetical study. Observational= Single-cohort observational study. POCT=Point-of-care Testing. TB=Tuberculosis. MDR=Multidrug-resistant tuberculosis. NR=not reported.
