# Supplementary material for: Impact of Molecular Diagnostics for Tuberculosis on Patient-Important Outcomes: A Systematic Review of Study Methodologies
Source: PLoS One. 2016 Mar 8;11(3):e0151073. doi: 10.1371/journal.pone.0151073 (PMC4783056; doi:10.1371/journal.pone.0151073)
Supplement: S4 Appendix — (PDF) [file pone.0151073.s004.pdf]

RCTs

	Random sequence generation	Allocation concealment	Blinding	Incomplete outcome data	Selective reporting	Other bias
Theron 2013	+	+	+	-	+	-
Mupfumi 2014	+	?	+	?	-	-
Cox 2014	+	-	+	+	+	-
Durovni 2014	+	-	-	-	+	+
Churchyard 2015	+	-	+	+	-	-

Pre/post implementation studies

	Patient sampling	Selection criteria	Exchangeability	Time trends	Incomplete outcome data
Boehme 2011	+	+	-	-	?
Skenders 2011	+	+	-	-	-
Hanrahan 2012	+	-	-	-	-
Yoon 2012	+	+	-	-	+
Jacobson 2012	+	+	-	+	+
Singla 2014	+	-	-	-	-
Naidoo 2014	+	-	-	+	+
Kipiani 2014	+	+	-	-	-
Cox 2015	+	-	-	+	+

Single-cohort hypothetical studies

	Patient sampling	Non-selective testing	Assumptions	Incomplete outcome data
Chaisson 2014	+	+	+	+
Sohn 2014	+	+	-	+
Lippincott 2014	+	+	+	-
Davis 2014	+	+	+	+

Single-cohort observational studies

	Patient sampling	Non-selective testing	Assumptions	Incomplete outcome data
Balcells 2012	+	+	-	+
Lyu2013	+	+	-	?
Van Rie2013	+	+	-	+
Hanrahan 2013	+	+	-	-
Kwak 2013	+	+	-	-
Ramirez 2014	+	+	-	?
Cohen 2014	+	-	-	-
