# Supplementary material for: Impact of Molecular Diagnostics for Tuberculosis on Patient-Important Outcomes: A Systematic Review of Study Methodologies
Source: PLoS One. 2016 Mar 8;11(3):e0151073. doi: 10.1371/journal.pone.0151073 (PMC4783056; doi:10.1371/journal.pone.0151073)
Supplement: S5 Appendix — (PDF) [file pone.0151073.s005.pdf]

“Regular” confounding

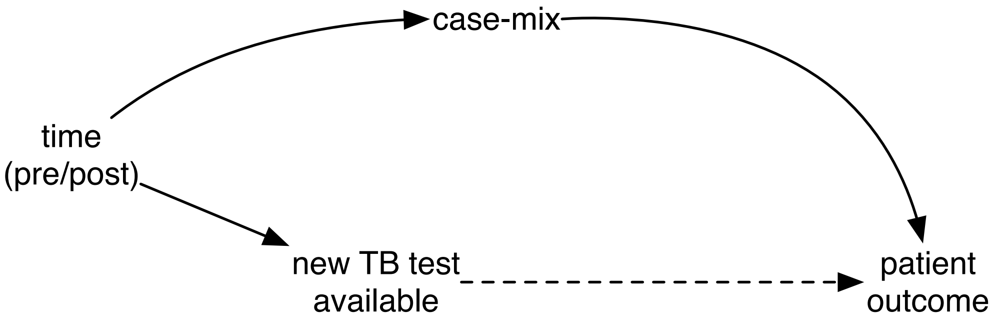

- case-mix may be e.g. socio-demographics, disease severity, co-morbidities
- resulting imbalances can be easily seen in a table comparing covariate-distribution
- can adjust, e.g. via regression

Confounding via time trends

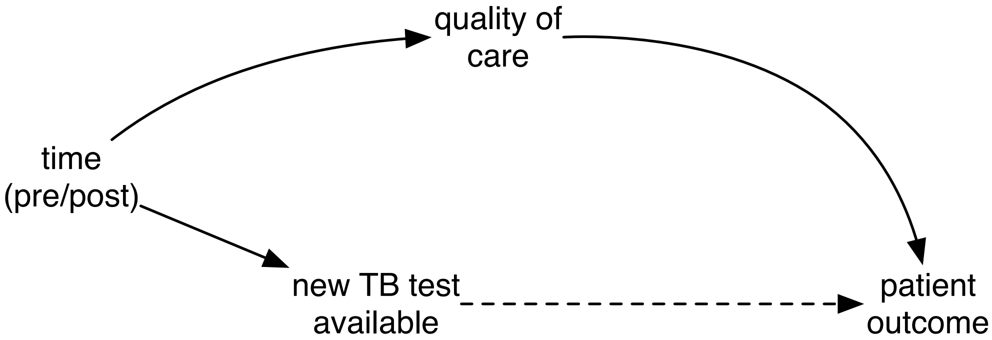

- quality of care may be e.g. availability of treatment for TB or HIV, decentralization, more rapid service provision
- usually this is not shown in a table but could be done
- usually this is not adjusted for but could be done, e.g. via regression

Confounding induced by selection bias

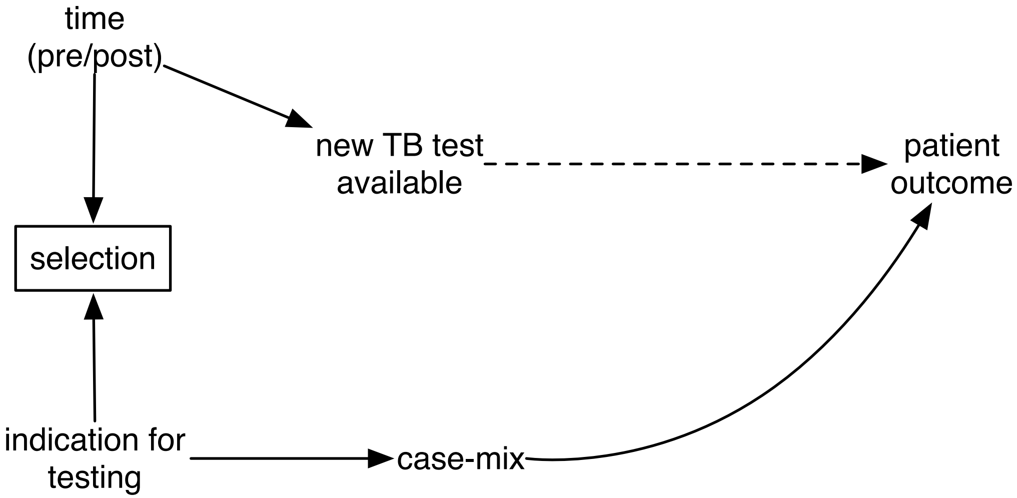

- may be introduced e.g. if indication for testing changes between pre/post (typically from “high-risk” patients to a broader group) and if the changed case-mix has different average patient outcomes
- this will be hard to adjust for as one would need data that is usually unavailable (e.g. complete resistance profile or other variables mediating the relationship between case-mix and outcomes); even if data is available, the positivity assumption may be violated
